# Investigation of Relationship between Occupational Stress and Cardiovascular Risk Factors among Nurses

**DOI:** 10.18502/ijph.v49i10.4699

**Published:** 2020-10

**Authors:** Amin SABERINIA, Anna ABDOLSHAHI, Saeed KHALEGHI, Yaser MORADI, Hossein JAFARIZADEH, Ali SADEGHI MOGHADDAM, Mohsen AMINIZADEH, Mehdi RAEI, Alireza KHAMMAR, Mohsen POURSADEQIAN

**Affiliations:** 1. Department of Emergency Medicine, School of Medicine, Shahid Beheshti University of Medical Sciences, Tehran, Iran; 2. Food Safety Research Center (Salt), Semnan University of Medical Sciences, Semnan, Iran; 3. Department of Nursing, School of Nursing & Midwifery, Alborz University of Medical Sciences, Karaj, Iran; 4. Patient Safety Research Center, Urmia University of Medical Sciences, Urmia, Iran; 5. Department of Nursing, School of Nursing & Midwifery, Dezful University of Medical Sciences, Dezful, Iran; 6. Vice Chancellor for Research and Technology, Kerman University of Medical Sciences, Religious Studies and Medicine Research Center, Kerman University of Medical Sciences, Kerman, Iran; 7. Health in Emergency and Disaster Research Center, University of Social Welfare and Rehabilitation Sciences, Tehran, Iran; 8. Health Research Center, Life Style Institute, Baqiyatallah University of Medical Sciences, Tehran, Iran; 9. Zabol Medical Plants Research Center, Department of Occupational Health, School of Health, Zabol University of Medical Sciences, Zabol, Iran; 10. Health Sciences Research Center, Torbat Heydariyeh University of Medical Sciences, Torbat Heydariyeh, Iran

**Keywords:** Cardiovascular diseases, Occupational stress, Nurses

## Abstract

**Background:**

One of the major causes of various work-related health problems among nurses is occupational stress. Hence, the main purpose of the present research was to find association between occupational stress of nurses and risk factors of cardiovascular disease.

**Methods:**

In this cross-sectional study, the Osipow job stress questionnaire was employed to assess the occupational stress among the 250 nurses in Emam Khomeini hospital of Tehran in 2018. Based on stress score for participants, subjects divided into two groups: Scoring of group one was 60–179 (mild and average stress) and group two between 180 and 300 (average to acute and acute stress). Systolic and diastolic blood pressures were recorded for subjects after 10hrs fasting. Then the blood samples were collected to measure cholesterol, triglyceride and glucose levels. For determining the association between education level, job experience and study groups, the Chi-square test and for comparing job stress between two groups of study the Mann–Whitney U test was used.

**Results:**

Subjects with job stress in group one was 70(28%) and group two was 180(72%). The association between level of education and two study groups was not significant (*P*=0.129) while between job experience and two study groups was significant (*P*=0.004). Mean of Blood glucose levels for group I (98.0± 37.5), was higher than group II (82.5±12.0) and statistically significant (*P*=0.001). No significant difference was found between two groups of study for other parameters.

**Conclusion:**

High level of work-related stress among subjects affected the values related to blood glucose level, but no significant relationship was found between other risk factors of cardiovascular diseases and occupational stress among nurses.

## Introduction

Variety of risk factors can influence the incidence of cardiovascular disease and its related high rates of mortality (1–3). Accordingly, most countries and health care organizations struggle with these disturbing diseases (4, 5).

Possibility of developing such disorders can rise under the influence of a variety of risk factors. The incidence rate of cardiovascular diseases is also influenced largely by these risk factors (6), resulting in higher mortality rate. Some of these factors are smoking, lack of physical activity, high blood pressure, weight gain, hyperinsulinemia and hypercholesterolemia (7, 8).

Stress caused by job status imposes physical and psychological tension due to lack of coordination between expectations of person and objective or cognitive demands of the jobs (9).

Chronic health problems such as cardiovascular diseases can occur because of occupational stress (10). Therefore, this issue has led researchers toward investigating the association between stress, including its occupational type, and risk factors of cardiovascular diseases (11, 12); numerous evidence in this regard are available, as well as on correlation between occupational stress and hypertension and heart disease (10, 13), or between psychosocial stress and cardiovascular diseases (14).

Nurses are at risk of stress related disease (9). Nurses Stress rose from busy working schedule, interprofessional conflict and their responsibilities and stress relationship with the disease is well documented (15,16). Stress situation is affected on burnout and quality of work of nurses (17,18). On the other hand, work condition has high effect on health. Iranian nurses have different situation from developed countries. Therefore, it’s maybe related to some disease. This study aimed to evaluate relationship between cardiovascular disease risk factors and job-related stress in stressed nurses. Understanding prevalence and underlying factors associated with such disorders could decrease spread of these disorders and planning prevention programs (10).

## Materials and Methods

This cross-sectional and descriptive-analytical study was carried out in 2018 on nurses in Imam Khomeni hospital of Tehran who had minimum three years of job experience as inclusion criteria.

The sample size was estimated at 250 with confidence level of 95% and error level of 5%. Demographic characteristics and job related stress information were collected through a questionnaire by interview. The occupational stress was assessed via Osipow job stress questionnaire (reviewed in 1998) with approved validity and reliability in other studies, consisting 60 questions in six subscales. The responses were scored as 5 points Likert scale from one to five. The questionnaire’s manual was employed to calculate total score and to interpret, as follows:

Scores of 60–119 for trivial stress, 120–179 for moderate stress, 180–239 for moderate to acute stress and 240–300 for acute stress. The population were assigned into two groups, the first group included trivial and trivial-moderate stress and the second group consisted of moderate-acute and acute stress according to the results of Osipow questionnaire (16).

To study the association between job experience and occupational stress, the data on educational level and job experience were collected using a questionnaire. The height and weight of the participants were also measured. Mean of the two consecutive tests was considered as the result. Time of the last meal before systolic and diastolic blood pressure determination was recorded. Then blood samples were collected to measure cholesterol, triglyceride and glucose levels using an automatic analyzer. Exclusion criteria were diabetes, history of heart diseases and history of taking medications for hypertension and hyperlipidemia (6).

For specifying data normality the Kolmogorov– Smirnov test was applied. To investigate the association between education level, job experience and study groups, the Chi-square test was used. According to the normality result the Mann– Whitney U test was done for comparing of job stress between two groups of study. All statistical analyses were performed using SPSS version 16.0 software (Chicago, IL, USA).

### Ethical Approval

Ethical consideration by University of Social Welfare and Rehabilitation Sciences ethical committee (code: IR.USWR.REC.1396.374) and plagiarism have been observed by the authors.

## Results

Overall, 30(12%) of participants had high school, 168(67.2%) had diploma, 33(13.2%) were with associate degree and 19(7.6%) had Bachelor degree. The job experience was 11–20 yr among 60(24%) of cases, 1–10 yr among 120(48%) of them and 20 yr or over among 70(28%) of subjects. The mean work hours was 14±4 hours and 59(23.6%) of participants were smokers. The biochemical tests results were as follows: cholesterol of 28 (11.2%) over 250 mg/dL and triglyceride level of 17 (6.8%) over 245 mg/dL, as well as systolic blood pressure of 11 (4.4%) over 145mmHg and diastolic blood pressure of 13 (5.2%) over 95mmHg.

Stress intensities among the study subjects according to OSIPOW job stress questionnaire to 10 (4%) participants was mild, to 60 (24%) persons were average, and to 165 (66%) was average to acute; accordingly, 70 (28%) participants were assigned to Group I and 128 (72%) to Group II (Table 1).

**Table 1:** Distribution of occupational stress prevalence in the nurses

***Stress intensity***	***Group study***	***n (%)***	***Stress intensity***	***n (%)***
Mild Stress and Average stress	One (I)	70 (28%)	Mild Stress	10 (4%)
			Average stress	60 (24%)
Average to acute stress and Acute stress	Two (II)	180 (72%)	Average to acute	165 (66%)
			Acute	15 (6%)
Total		250 (100%)		250 (100%)

According to Table 2 no significant association was found between educational level and job stress (*P*=0.219), while between job stress and job experience the association was significant (*P*=0.004) based on the results of Chi-square test.

**Table 2:** Cross tabulation of education level, job experience and study groups

***Parameter***	Group I^*^	Group II^**^	***P-value***
Level of education	n (%)	n (%)	
High school	13 (18.57)	17(9.44)	0.129
Diploma	47(67.15)	121(67.22)	
Associate degree	7(10)	26(14.44)	
Bachelor degree	3(4.28)	16(8.90)	
Job experience			
1–10	25 (35.71)	95(52.78)	0.004
11–20	15(20.43)	45(25)	
>20	30(42.86)	40(22.22)	

* Trivial and trivial-average stress

** Average-acute and acute stress

Table 3 shows smoking rate per day between two groups was not significance (*P*=0.719). The blood glucose levels for group I was 98.0± 37.5 and for group two was 82.5±12.0, that difference between them statistically was significant (*P*=0.001). However the difference of blood Triglycerides levels between two groups was not significant (*P*=0.215), as well as for blood cholesterol levels (*P*=0.405), diastolic blood pressure (*P*=0.380) and systolic blood pressure (*P*=0.213).

**Table 3:** Comparison of cardiovascular risk factors among study groups

***Risk factor***	Group I ^*^	Group II ^**^	P-value ^***^
Smoking rate (per day)	2.36±15.2	3.07±10.41	0.719
Blood glucose levels	98.0± 37.5	82.5±12.0	0.001
Blood Triglycerides levels	178.44±106.8	160.5±88.9	0.215
Blood Cholesterol levels	187.41±37.43	182.80±43.21	0.405
Diastolic blood pressure	78.87±13.52	77.28±10.7	0.380
Systolic blood pressure	120.17±18.22	117.18±12.81	0.213

* Trivial and trivial-average stress

** Average-acute and acute stress

*** From Mann–Whitney U test

## Discussion

Results demonstrated a relatively high rate of job stress as 75.5% of subjects were suffering from different stress levels. Smoking had no significant association with the level of stress perceived by the subjects (20). Results indicated a significant correlation between job stress and blood glucose level.

No association was found among work-related stress on the one hand and educational level and work experience on the other hand. Work experience had stronger correlation with work-related stress compared to stress and CVD risk factors, except for glucose. Due to the inconsistency among the results, further investigations are required in this field. Blood glucose had significant relationship job stress but no relationship between stress and other CVD risk factors (21), although a study evaluated association between occupational stress and risk factors of cardiovascular disease in locomotive operators(6), however, stress related to high level of cholesterol in locomotive operators despite our study. Approximately 23% of subjects of a study entitled “Healthy Blood” conducted in 1997 in Tehran, Iran, showed high diastolic blood pressure (22).

The findings obtained from the current research are less reliable because of its cross-sectional nature in comparison to the other studies considered the long-term study durations. Hence, we cannot generalize our results and not be able to accurately justify the non-significant correlations between work-related stress and CVD risk factors. Similar studies are performed to take into account the long-term durations for understanding the true relationship and to obtain authentic results.

## Conclusion

Most nurses as a study unit in our study were suffering from high occupational stress. Despite this issue, the results showed no significant relationship between work-related stress and cardiovascular disease risk factors except blood glucose. The cross-sectional nature of our study and young age of the participants on average were leading causes affecting our results.

## Ethical considerations

Ethical issues (Including plagiarism, informed consent, misconduct, data fabrication and/or falsification, double publication and/or submission, redundancy, etc.) have been completely observed by the authors.

## References

[1] American Heart Association (2005) Heart disease and stroke statistics Update 2005. https://my.clevelandclinic.org/ccf/media/files/he art/1105390918119HDSStats2005Update.pdf . Accessed 01 Sept 2010.

[2] Raymond I Pedersen F Steensgaard-Hansen F (2003). Prevalence of impaired left ventricular systolic function and heart failure in a middle aged and elderly urban population segment of Copenhagen. Heart, 89 (12): 1422– 1429. 14617553 10.1136/heart.89.12.1422 PMC1767972

[3] Roger VL Weston SA Redfield MM (2004). Trends in heart failure incidence and survival in a community-based population. JAMA, 292 (3): 344– 50. 15265849 10.1001/jama.292.3.344

[4] O'connell J (2000). The economic burden of heart failure. Clin Cardiol, 23: III6–10. 10754775 10.1002/clc.4960231503 PMC6654942

[5] Stewart S MacIntyre K Capewell S McMurray J (2003). Heart failure and the aging population: an increasing burden in the 21st century? Heart, 89 (1): 49– 53. 12482791 10.1136/heart.89.1.49 PMC1767504

[6] Shahbazi A Rahmani N Abbasi M (2018). Association between Occupational Stress and Risk Factors of Cardiovascular Disease in Locomotive Operators. Iranian Heart Journal, 19 (2): 20– 26.

[7] Pyörälä M Miettinen H Halonen P (2000). Insulin resistance syndrome predicts the risk of coronary heart disease and stroke in healthy middle-aged men. Arterioscler Thromb Vasc Biol, 20 (2): 538– 44. 10669654 10.1161/01.atv.20.2.538

[8] Williams MA Petratis MM Baechle TR (1987). Frequency of physical activity, exercise capacity, and atherosclerotic heart disease risk factors in male police officers. J Occup Med, 29( 7): 596–600. 3612337

[9] Poursadeghiyan M Moghimian M Amjad RN (2017). Effects on job stress on Iranian clinical nurses. Ann Trop Med Publ Health, 10 (4): 985– 988.

[10] Kaplan JR Chen H Manuck SB (2009). The relationship between social status and atherosclerosis in male and female monkeys as revealed by meta-analysis. Am J Primatol, 71 (9): 732– 41. 19452517 10.1002/ajp.20707 PMC3366586

[11] Mariammal T Jaisheeba AA Sornaraj R. (2012). Work influenced occupational stress and cerebrovascular risk among teachers and office workers. Journal of Chemical and Pharmaceutical Research, 4 (3): 1807– 1811.

[12] Cohen S Janicki-Deverts D Miller GE (2007). Psychological stress and disease. JAMA, 298 (14): 1685– 7. 17925521 10.1001/jama.298.14.1685

[13] Pickering T Devereux R James G (1996). Environmental influences on blood pressure and the role of job strain. J Hypertens Suppl, 14 (5): S179– 85. 9120676

[14] Hemingway H Marmot M (1999). Psychosocial factors in the aetiology and prognosis of coronary heart disease: systematic review of prospective cohort studies. BMJ, 318: 1460. 10346775 10.1136/bmj.318.7196.1460 PMC1115843

[15] Soltaninejad M Khammar A Aminizadeh M (2020). Shift working disorders among nurses of Tehran hospital and its related factors in 2016. Work. 66( 1): 213–219. 32417828 10.3233/WOR-203165

[16] Poursadeghiyan M Abbasi M Mehri A (2016). Relationship between job stress and anxiety, depression and job satisfaction in nurses in Iran. The Social Science, 11 (9): 2349– 2355.

[17] Khammar A Dalvand S Hashemian AH (2018). Data for the Prevalence of Nurses' Burnout in Iran (A Meta-Analysis dataset). Data Brief, 20: 1779–1786 30294624 10.1016/j.dib.2018.09.022 PMC6169446

[18] Khammar A Poursadeghiyan M Marioryad H (2019). Patient Safety Climate and Its Affecting Factors Among Rehabilitation Health Care Staff of Hospitals and Rehabilitation Centers in Iran-Tehran. IRJ, 17 ( 1) : 39– 48

[19] Kouvonen A Kivimäki M Virtanen M Pentti J Vahtera J (2005). Work stress, smoking status, and smoking intensity: an observational study of 46 190 employees. J Epidemiol Community Health, 59 (1): 63– 69. 15598729 10.1136/jech.2004.019752 PMC1763376

[20] Pelfrene E De Backer G Mak R (2002). Job stress and cardiovascular risk factors. Results from the BELSTRESS study. Arch Public Health, 60: 245– 268.

[21] Biglari H Ebrahimi MH Salehi M (2016). The Relationship of Occupational Stress to Cardiovascular Disease Risk Factors in Drivers. Int J Occup Med and Environ Health, 29 (6): 895– 901. 27869240 10.13075/ijomeh.1896.00125

[22] YadegarfarGh A Gharaaghajiasl R Allahyari T Sheikhbagloo R (2010). Assessing the Relationship between Occupational Stress and Risk factors of cardiovascular in the Urmia Petrochemical Company employees. Journal of Isfahan Medical School, 28 (112): 645– 60.

